# Commentary: RNA transcripts in salivary extracellular vesicle cargo isolated from aged populations

**DOI:** 10.3389/fragi.2026.1799802

**Published:** 2026-03-24

**Authors:** Ling Yin

**Affiliations:** 1 College of Life Science, Weifang Institute of Technology, Weifang, Shandong, China; 2 Weill Cornell Medicine, Cornell University, New York, NY, United States

**Keywords:** aging, neurodegenerative disorders, non-invasive biomarkers, RNA transcripts, salivary extracellular vesicle

## Introduction

The increasing prevalence of neurodegenerative disorders demands the development of accessible, non-invasive diagnostic tools. The study by Wen et al. makes a timely contribution by thoroughly characterizing the RNA content of salivary extracellular vesicles (salEVs) from cognitively healthy older adults, positioning saliva as a promising biofluid for biomarker discovery (Wen et al., 2026). Saliva diagnostics, as reviewed by Kaczor-Urbanowicz et al., offers inherent advantages of being fast, easy, inexpensive, and non-invasive to collect (Kaczor-Urbanowicz et al., 2017). This commentary will discuss the key findings of Wen et al., integrating their results with the cited literature to highlight the study’s significance, mechanistic insights, and future translational potential.

## Comparative advantage of saliva and mechanistic plausibility

A central finding by Wen et al. is the higher abundance of neurodegeneration-associated RNA transcripts in salEVs compared to blood-derived EVs (bEVs) (Wen et al., 2026). This suggests saliva may be a superior medium for neurological biomarker discovery. The clinical appeal is evident, as saliva collection is more acceptable for patients and suitable for longitudinal monitoring. The detection of CNS-relevant transcripts in saliva gains plausibility from established research on extracellular vesicle (EV) biology. Banks et al. demonstrated that EVs can traverse the blood-brain barrier (BBB) via specific transport mechanisms, with inflammatory states modulating their kinetics (Banks et al., 2020). This provides a critical pathway for brain-derived molecular signatures to reach peripheral biofluids. Wen et al. further suggest active RNA packaging into salEVs, implied by enriched transcription factor binding motifs, indicating these vesicles may reflect the pathophysiological state of their cells of origin (Wen et al., 2026).

## Retrotransposon transcripts: a link to neurodegenerative mechanisms

One of the most intriguing observations is the detection of retrotransposon-derived transcripts (LINE, SINE, LTR) within salEVs (Wen et al., 2026). This finding resonates deeply with contemporary understanding of neurodegenerative pathogenesis. Frost and Dubnau comprehensively reviewed the role of retrotransposons and endogenous retroviruses, highlighting their activation as a contributing factor in age-related disorders involving TDP-43 and tau pathologies (Frost et al., 2024). The retrotransposon-encoded products can trigger innate immune responses, leading to neuroinflammation. This concept is mechanistically supported by De Cecco et al., who showed that L1 retrotransposon activity in senescent cells drives a type-I interferon response, promoting age-associated inflammation or “inflammaging” (De Cecco et al., 2019). Their work demonstrated that inhibiting L1 reverse transcriptase reduced inflammation in aged mice. Therefore, the presence of these transcripts in salEVs from older adults could serve as a biomarker for the inflammatory aging milieu, a known risk factor for neurodegeneration, potentially offering a window into pre-symptomatic pathological processes.

## Validation from related fields and specificity of approach

The diagnostic potential of salEVs for neurological conditions is not without precedent. Cheng et al. provided compelling evidence that salEV gene expression profiles could distinguish patients with mild traumatic brain injury (TBI) from healthy controls (Cheng et al., 2019). Notably, they identified upregulated genes associated with Alzheimer’s disease pathways in TBI patients, suggesting overlapping molecular mechanisms between acute injury and chronic neurodegeneration (Cheng et al., 2019). This independent validation strengthens the rationale for exploring salEVs in diseases like AD and PD. Furthermore, Wen et al. addressed a major confounder in saliva research—microbial RNA contamination—by employing ultracentrifugation to isolate EVs, thereby enriching for human-derived RNA and enhancing biomarker specificity (Wen et al., 2026). This methodological rigor aligns with the need for precision in saliva diagnostics (Kaczor-Urbanowicz et al., 2017).

## The significance of lncRNA signatures and broader context

The study’s analysis of long non-coding RNA (lncRNA) content in salEVs reveals a selective packaging mechanism, where specific lncRNAs like PCBP1-AS1 are enriched over highly abundant cellular ones like MALAT1 (Wen et al., 2026). This pattern suggests that the lncRNA signature within salEVs may carry specific information about cellular state. The broader principle that specific lncRNAs can serve as potent disease biomarkers was established in oncology by Ji et al., who identified MALAT1 as a prognostic marker in early-stage non-small cell lung cancer (Ji et al., 2003). This foundational work supports the interpretation that the specific lncRNA profile in salEVs may similarly encode valuable diagnostic or prognostic information for neurodegenerative diseases. The research by Wen et al. is aptly contextualized within the expanding field of liquid biopsy. As reviewed by Bazan Russo et al. in the context of precision oncology, the analysis of circulating biomarkers, including EVs, provides a dynamic, minimally invasive approach to managing disease heterogeneity and treatment response (Bazan Russo et al., 2025). The challenges and opportunities described in oncology liquid biopsy—such as early detection, monitoring minimal residual disease, and assessing therapeutic resistance—directly parallel the needs in neurodegenerative disease management, underscoring the transformative potential of salEV-based approaches.

## Discussion and future directions

Wen et al. have constructed a comprehensive and compelling framework that integrates EV biology, transcriptomics, and clinical neuroscience. The study effectively argues that saliva, through salEV analysis, is a viable and superior biofluid for discovering neurodegeneration biomarkers. However, the authors appropriately note limitations, including the cross-sectional design and modest sample size of cognitively healthy individuals. Future research must prioritize longitudinal studies in large, diverse cohorts that include individuals across the spectrum of cognitive decline and specific neurodegenerative diagnoses. Such studies are essential to determine whether salEV RNA profiles can predict disease onset, differentiate between disorders, or track progression.

Additional questions remain. Further investigation is needed to clarify the precise cellular origins of the salEVs carrying these RNA signatures. While a CNS contribution is plausible, contributions from salivary glands, oral tissues, or peripheral immune cells must be defined to accurately interpret biomarker signals. Standardizing salEV isolation, RNA extraction, and analysis protocols across laboratories will be another critical step toward clinical translation.

## Conclusion

In summary, the study by Wen et al. represents a significant advance by harnessing the accessibility of saliva and the information-rich cargo of EVs. By thoughtfully integrating findings with established knowledge—from EV trafficking across the BBB and the role of retrotransposons in neurodegeneration to prior validation in TBI, foundational lncRNA biomarker principles, the framework of saliva diagnostics, and the promise of liquid biopsy—the authors present a coherent and persuasive case for salEV RNA as a non-invasive window into neurodegeneration. This work lays a solid foundation for future research aimed at translating these promising biomarkers into clinical tools for early detection, stratification, and monitoring, potentially transforming the management of debilitating neurodegenerative diseases.
