# Correction: Commentary: RNA transcripts in salivary extracellular vesicle cargo isolated from aged populations

**DOI:** 10.3389/fragi.2026.1838683

**Published:** 2026-04-01

**Authors:** Ling Yin

**Affiliations:** 1 College of Life Science, Weifang Institute of Technology, Weifang, Shandong, China; 2 Weill Cornell Medicine, Cornell University, New York, NY, United States

**Keywords:** aging, neurodegenerative disorders, non-invasive biomarkers, RNA transcripts, salivary extracellular vesicle

Affiliation “College of Medicine, Weifang Institute of Technology, Weifang, Shandong, China” was erroneously given for author Ling Yin. The correct affiliation is “College of Life Science, Weifang Institute of Technology, Weifang, Shandong, China”.

The original article has been updated.

